# Phylogeny of coccidian parasites (Eimeriorina, Apicomplexa) infecting shrews

**DOI:** 10.1093/femsmc/xtag047

**Published:** 2026-09-04

**Authors:** Anna Mácová, Roman Hrdlička, Jana Kvičerová

**Affiliations:** Department of Parasitology, Faculty of Science, University of South Bohemia, Branišovská 1760, 370 05 České Budějovice, Czech Republic; Department of Parasitology, Faculty of Science, University of South Bohemia, Branišovská 1760, 370 05 České Budějovice, Czech Republic; Department of Parasitology, Faculty of Science, University of South Bohemia, Branišovská 1760, 370 05 České Budějovice, Czech Republic

**Keywords:** Soricidae, *Eimeria*, diversity, host specificity, oocyst morphology, phylogenetic relationships

## Abstract

Despite their ecological importance and protected status, the diversity, host associations, and evolutionary relationships of coccidia infecting shrews (Eulipotyphla: Soricidae) remain poorly understood. In particular, the extent of host specificity, the occurrence of host-switching events, and the relationships among coccidian lineages infecting different shrew hosts remain unclear. Here, we investigated coccidian diversity and phylogenetic relationships across several shrew genera and species to clarify parasite–host associations and provide new insights into the evolutionary processes shaping coccidian diversity in shrews. Fecal samples from 188 shrews representing the genera *Crocidura, Neomys, Sorex*, and *Suncus*, collected across multiple localities in Europe and two localities in Asia, were examined. Coccidia were detected in one quarter of the samples, generally at low intensities. Phylogenetic analyses based on partial COX1, COX3, and 18S rRNA sequences identified four distinct clusters: three were host-specific (two associated with *Crocidura* and one with *Neomys*), whereas one lineage was shared among *Crocidura, Neomys*, and *Sorex*. Two *Isospora* sequences from *Sorex araneus* clustered with isosporans previously reported from moles. No clear geographic structuring was observed, and the phylogenetic structure also did not mirror the observed morphological characters. The results indicate that host switching and subsequent repeated dispersal occur in eimerians of shrews, suggesting that these processes may be more widespread across small-mammal hosts than previously recognized. The findings expand current knowledge on coccidian diversity in shrews and provide new insights into the host specificity, ecology, and evolutionary dynamics of the parasitic protists.

## Teaser

Fecal analyses of European and Asian shrews uncovered four coccidian lineages with varying host specificity and evidence of host switching, revealing unexpectedly complex evolutionary and ecological dynamics in shrew eimerians.

## Introduction

Coccidia are protists infecting a broad range of domestic and wild-living hosts. In general, most studies have focused on species that threaten human health (e.g. *Plasmodium, Toxoplasma*) or infect farm animals such as poultry, rabbits, or cattle (mainly genus *Eimeria*). These studies have mainly addressed their pathogenicity, life cycles, and oocyst morphology (Kreier and Baker 1987, Barta et al. 1997, Duszynski and Upton 2001, Pakandl 2009, Vrba and Pakandl 2014, Hinsu et al. 2018, Bangoura et al. 2022). In the recent era of molecular biology, studies on evolutionary and host–parasite relationships have greatly expanded and become a common part of taxonomic descriptions (Morrison et al. 2004, Kvičerová and Hypša 2013, Vrba and Pakandl 2015, Mácová et al. 2018, Trefancová et al. 2019, 2021). In light of molecular genetics, these studies have revealed that not all previously recognized concepts based only on morphology of oocysts are valid. In eimerians, oocyst morphology has shown to be a phylogenetically inconsistent trait. The genus *Eimeria* is polyphyletic, and other genera (e.g. *Caryospora, Cyclospora*, and some *Isospora* species) cluster within *Eimeria* (Morrison et al. 2004). For a long time, rodent-infecting species of *Eimeria* were thought to form two phylogenetic clusters based on the presence/absence of an oocyst residuum (OR) in their oocysts (Zhao and Duszynski 2001, Power et al. 2009). However, it was later revealed that *E. myoxi* formed its own cluster, i.e. a third cluster (Kvičerová et al. 2011). Recently, it has been demonstrated that splitting *Eimeria* into three clusters may be an artefact depending on the number of examined and analyzed samples in the dataset (Mácová et al. 2018). Another objection to taxonomy based solely on morphological features is posed by changes in oocyst size, and the formation of inner structures during sporulation and patency (Long and Joyner 1984, Parker and Duszynski 1986, Gardner and Duszynski 1990).

With a few exceptions (Matsubayashi et al. 2005, Kvičerová et al. 2011, Mácová et al. 2018, Trefancová et al. 2019, 2021, Kubiski et al. 2022), studies on coccidian phylogeny and parasite–host relationships in wild-living hosts are scarce, and no studies have investigated this trait in shrews. This is likely because shrews are protected by conservation laws, making sample collection difficult. Until now, the vast majority—if not all—of the publications concerning eimerians of insectivores have focused on oocyst morphology, pathogenicity, and life cycles (Yakimoff and Gousseff 1935, Golemansky and Yankova 1973, Hertel and Duszynski 1987, Duszynski and Upton 2000, Milek and Seville 2003, McAllister et al. 2021). Some of the older studies are uninformative, as the morphological descriptions are incomplete or simple, and they lack molecular data. Similarly, basic taxonomical and prevalence data are scarce. Only a small fraction of insectivorous species (about 9% of nearly 500 species) have been screened for the presence of coccidia: ~80 species of coccidia have been described and named, 10 of them from European shrews (Lynch and Duszynski 2008), which represents only a small number of the coccidians that may potentially infect these hosts.

The aim of this study was to analyze fecal samples from shrews that were accidentally obtained over the past several years, and to explore the phylogenetic relationships of their coccidian parasites. By studying coccidia and their hosts—the shrews—this manuscript further contributes to a broader understanding of host-parasite relationships. This study represents the first and complex view on the phylogenetic relationships of coccidia infecting insectivores, and resolves some of the confusion and questions associated with this group of parasites.

## Materials and methods

### Origin of the hosts and parasites, microscopical examination

**Figure 1:**
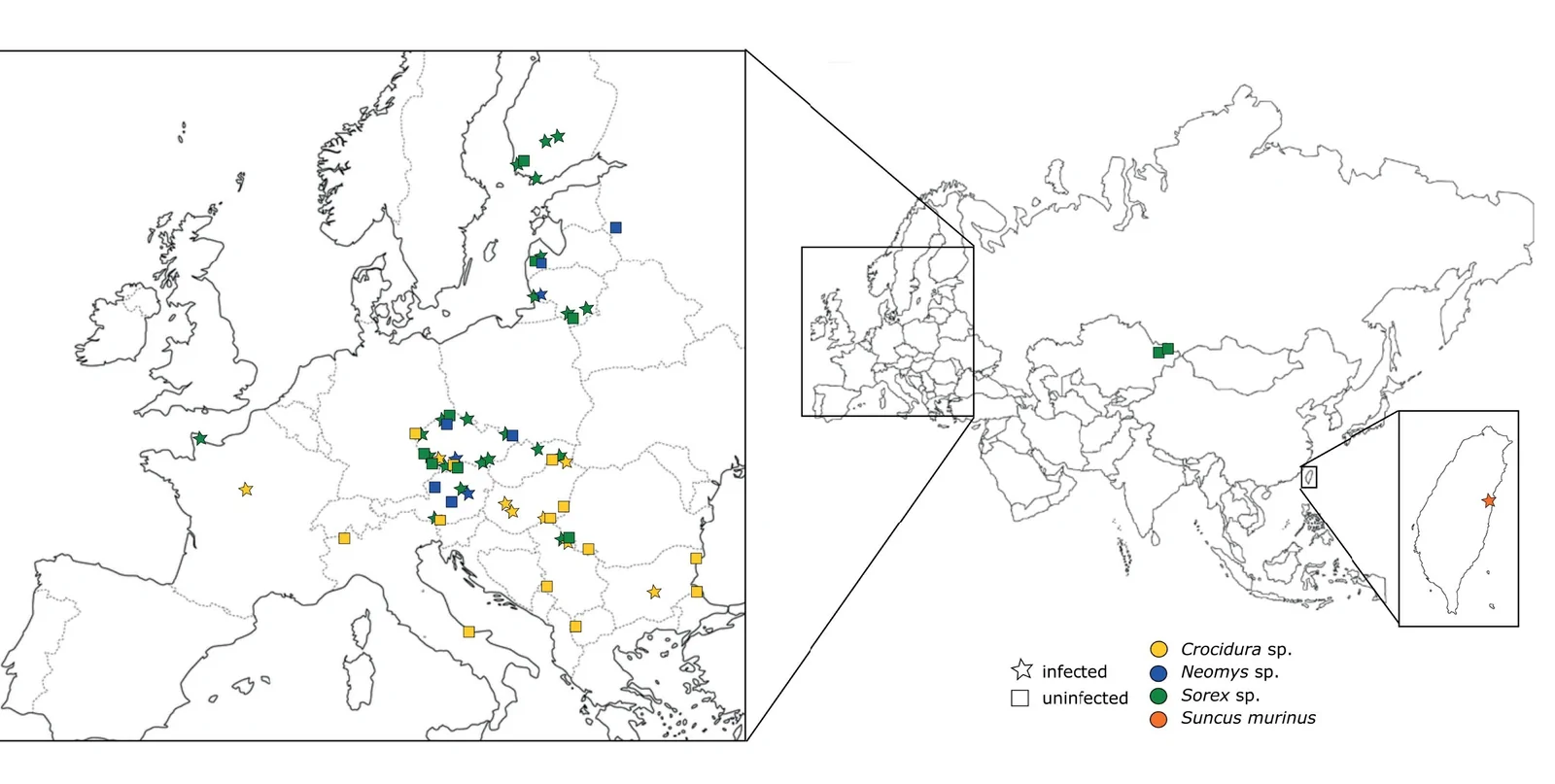
Map of sampled specimen. A map showing the collected and examined genera of shrews (marked with colored symbols), infected and uninfected with coccidia (marked with an asterisk and a square, respectively).

**Figure 2:**
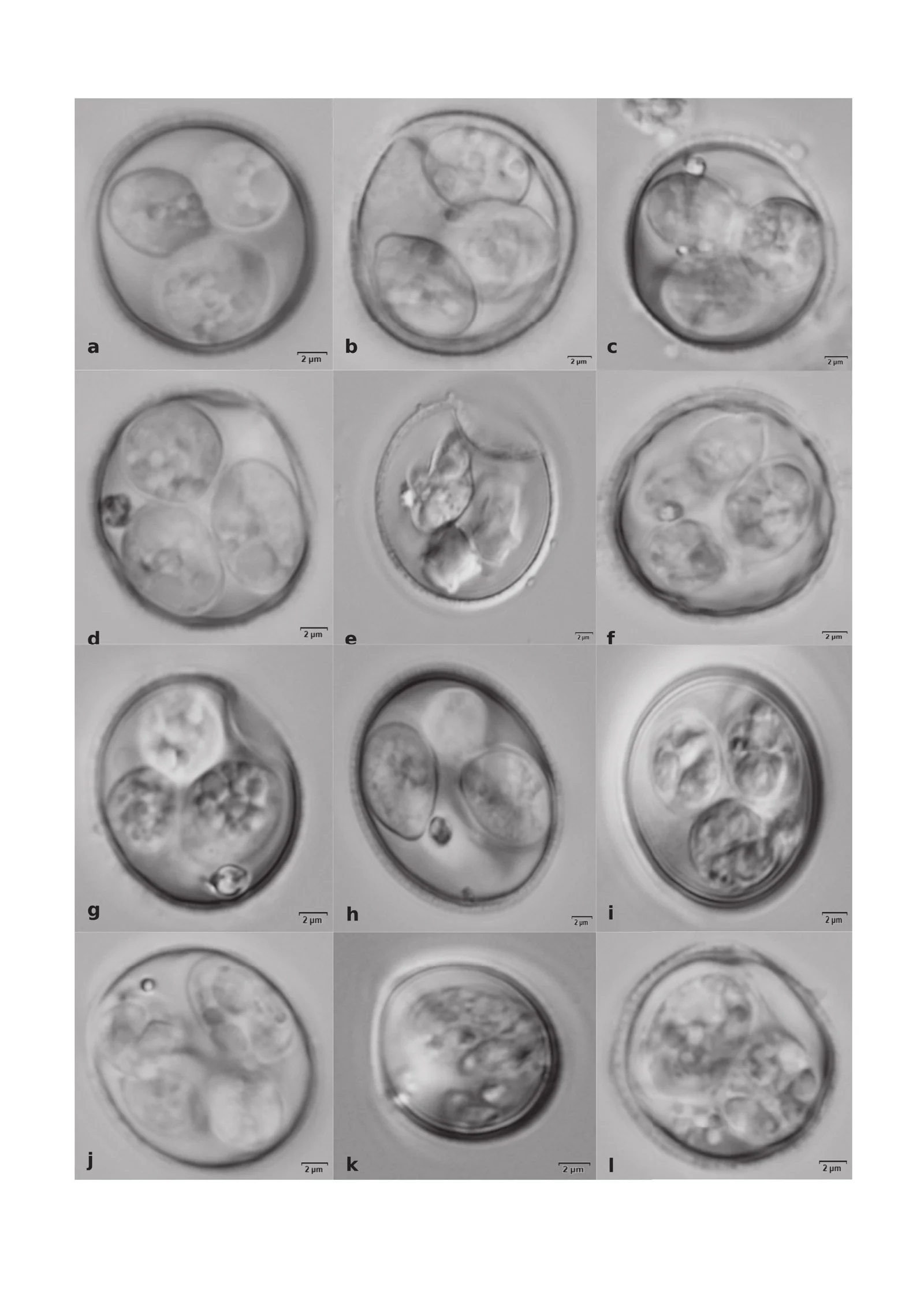
Photomicrographs of sporulated oocysts of coccidia observed in this study. Scale bar 2 μm. (a–f) *Eimeria* spp. group “Croci 1”: (a) isolate 23 696 (*Crocidura leucodon*), note rough oocyst wall and granular sporocyst residuum; (b) isolate B4B2 (*Crocidura* sp.), note rough oocyst wall, conspicuous Stieda body, and granular sporocyst residuum; (c) isolate 54STR (*C. suaveolens*), note rough oocyst wall, presence of the polar granule, and granular sporocyst residuum; (d) isolate 34RUM (*C. suaveolens*), note large and distinct polar granule and granular sporocyst residuum; (e) isolate 6HB3 (*C. leucodon*), note rough oocyst wall and the polar granule; (f) isolate 8HB1 (*Crocidura* sp.), note ellipsoidal polar granule and distinct Stieda body. (g–i) *Eimeria* spp. group “Croci 2”: (g) isolate 23 706 (*C. leucodon*), note large and distinct polar granule and granular sporocyst residuum; (h) isolate TW4 (*Suncus murinus*), note rough oocyst wall, two distinct polar granules, and distinct Stieda body; (i) isolate 19CR (*C. russula*), note smooth oocyst wall. (j–k) *Eimeria* spp. group “Neomys”: (j) isolate NF58 (*Neomys fodiens*), note the presence of small spherical polar granule; (k) isolate 60R (*N. anomalus*), note smooth oocyst wall. (l) *Isospora* sp. (isolate 21 032, So. araneus), note rough and thick oocyst wall.

Shrews are protected animals, therefore, the samples used in this study originated exclusively from individuals found dead on paths and roads in 14 European and 2 Asian countries during 2007–2018 (Fig. 1). The carcasses were collected and dissected, and the faeces together with a part of the digestive tract were preserved in 2.5% (w/v) potassium dichromate (K_2_Cr_2_O_7_) solution (according to Williams et al. 2010). The fecal samples were processed using density-gradient flotation in Sheather’s sucrose solution (specific gravity 1.30) (Zajac and Conboy 2006, Smith et al. 2007). Determination of the coccidian species/morphotypes was based on the morphology and morphometry of sporulated oocysts (see Fig. 2, Tables 1 and S3), following guidelines published by Duszynski and Wilber (1997) and Berto et al. (2014). An Olympus BX53 light microscope (Olympus, Tokyo, Japan) equipped with a DP-73–1-51 high-resolution cooled digital camera (Olympus), and Olympus cellSens Standard v1.13 imaging software were used.

**Table 1:** Morphological features of oocysts in coccidia-positive samples, ordered by oocyst size.

Sample code	Oocyst size [µm]	Range of the oocyst size [µm]	L/W	Oocyst wall [µm]	Sporocyst size [µm]	Range of the sporocyst size [µm]	SB	PG	SR	RB	Note
**60R**	13.9 × 11.8	13.6–14.3 × 11.5–12.2	1.2	na	7.7 × 5.9	7.2–8.2 × 5.7–6.1	?	?	?	?	PS
**23 706**	15.9 × 14.5	15.2–16.3 × 13.8–15.0	1.1	0.7	8.2 × 7.0	7.8–8.8 × 6.1–7.4	?	1	1?	?	PS
**RL**	16.8 × 14.6	16.4–17.3 × 14.2–14.9	1.2	?	na	na	?	?	?	?	US
**23 696**	16.8 × 15.3	15.4–19.9 × 13.9–16.9	1.1	0.8	8.8 × 6.5	6.9–10.7 × 5.4–7.6	+	1	+	+	
**21 032**	17.3 × 15.2	16.1–19.3 × 12.9–16.4	1.1	1.0	7.7 × 5.2	7.3–10.8 × 4.7–9.2	?	1	?	?	
**NF58**	17.4 × 15.5	16.3–19.1 × 14.2–16.6	1.1	0.7	8.7 × 5.9	6.6–9.9 × 4.7–7.0	+	1 (more?)	+?	+?	
**19CR**	17.9 × 16.8	15.7–19.9 × 15.1–18.9	1.1	0.7	8.3 × 5.9	7.1–10.0 × 5.5–6.8	+ flat	1	?	+	
**34RUM**	18.1 × 16.5	16.6–19.0 × 15.8–17.7	1.1	0.9	10.1 × 6.8	8.6–12.1 × 6.3–7.7	+	1	+	+	
**54STR**	18.3 × 17.8	17.3–19.8 × 16.1–19.7	1.0	1.0	8.5 × 7.1	6.3–10.8 × 6.2–8.3	+	1	+?	+	PS
**SA136**	19.3 × 17.8	18.1–20.5 × 16.5–20.0	1.1	0.8	?	?	?	?	?	?	PS
**TW4**	20.0 × 17.7	17.0–22.9 × 15.7–19.3	1.1	1.0	9.4 × 6.8	7.6–10.9 × 5.7–8.1	+ point	+	?	+	
**8HB1**	20.9 × 18.8	19.1–22.9 × 15.9–20.9	1.1	1.2	9.4 × 6.5	6.6–11.9 × 5.0–8.5	+	1 (1–2?)	+?	+?	
**TW1**	21.6 × 21.6	na	1.0	1.1	9.4 × 7.3	na	?	+	?	?	US
**6HB3**	21.8 × 21.1	21.4–22.5 × 20.8–21.5	1.1	0.8	11.1 × 7.0	9.6–14.7 × 5.7–7.8	?	?	?	?	
**B4B2**	22.1 × 19.4	20.9–22.7 × 18.7–20.1	1.1	1.2	10.4 × 7.7	8.1–11.8 × 5.6–9.4	+	more?	?	?	PS
**6HB1**	23.2 × 19.5	21.7–24.9 × 18.3–21.0	1.2	1.1	?	?	?	?	?	?	PS

L/W, length/width ratio; SB, Stieda body; PG, polar granule; SR, sporocyst residuum; RB, refractile body; na, data not available; ?, unclear; PS, partially sporulated; US, unsporulated; and +, present.

### DNA extraction, PCR amplification, sequencing

Genomic DNA was extracted from the coccidia-positive fecal samples using the FastDNA^®^ SPIN Kit for Soil (MP Biomedicals, LLC, Santa Ana, CA, USA), following the manufacturer’s protocol. A polymerase chain reaction (PCR) amplification was performed with Coccidia-specific primers targeting the gene encoding the small subunit of 18S rRNA (SSU), and the mitochondrial genes for cytochrome c oxidase subunit I (COX1) and III (COX3). Primers for 18S rRNA, COX1, and COX3 were adopted from Kvičerová et al. (2008), Schwarz et al. (2009), and Ogedengbe et al. (2015), respectively. HotStarTaq DNA Polymerase (Qiagen, Hilden, Germany) was used for all PCR reactions. PCR products of expected sizes were enzymatically purified using alkaline phosphatase and exonuclease I (Thermo Fisher Scientific, Waltham, MA, USA) and sequenced in both directions via the Sanger method by Macrogen, Inc. (Amsterdam, The Netherlands) on an automated 3730XL DNA analyzer.

### Sequence assembling, alignments, phylogenetic analyses

Obtained sequences were verified by the BLAST algorithm (https://blast.ncbi.nlm.nih.gov/Blast.cgi), edited and assembled using the Sequence Scanner v1.0 (Applied Biosystems), EditSeq v5.05, and SeqMan v5.05 (DNASTAR, Inc., Madison, WI, USA) programs, and deposited in the NCBI GenBank database under the accession numbers provided in Table S1. The final dataset included the obtained sequences together with relevant reference sequences from the NCBI GenBank database (for more details, see Table S1). Alignments were created in Geneious v9.1.3 (http://www.geneious.com; Kearse et al. 2012) using the MAFFT v1.3.6 algorithm with default parameters (Katoh and Standley 2013), and then manually adjusted and trimmed. The 18S rRNA sequences were aligned in nucleotide mode; COX1 and COX3 sequences were aligned in the amino acid mode, then switched to nucleotide mode and used in the analyses. A concatenated matrix was assembled in SeaView (Gouy et al. 2010).

Phylogenetic relationships were reconstructed by Bayesian inference, and maximum likelihood approaches. Bayesian inference was performed in MrBayes v3.2 (Huelsenbeck and Ronquist 2001), with the Markov Chain Monte Carlo (MCMC) run for 10 million generations and tree sampling every 100 generations; the trees were summarized after removing 25% burn-in. Maximum likelihood was carried out in PhyML v3.1 (Guindon and Gascuel 2003), with branch support assessed via 1000 replicates. Using jModeltest (Posada 2008, 2009), the GTR + Г + I was selected as the best-fitting model of evolution. Final trees were visualized and exported using TreeView v1.6.6 (Page 1996) and Inkscape v0.91 (https://inkscape.org).

## Results

Fecal samples were microscopically analyzed from 188 shrew individuals (124 *Sorex* spp., 37 *Crocidura* spp., 24 *Neomys* spp., and 3 *Su. murinus*). Of these, 45 (24%) were coccidia-positive. Because the animals were found at various stages of decay, and some were provided by amateurs who could identify only the genus, not all individuals were determined to species level. The lowest prevalence of coccidia was detected in *Sorex*; only 11 of 124 (9%) sampled individuals were infected, and they mostly contained only a few oocysts. Ten of 24 (42%) sampled individuals of *Neomys* spp. contained coccidian oocysts; however, the intensity of infection was also very low. A considerably higher prevalence was observed in *Crocidura*, where 21 of 37 (57%) samples were infected with coccidia, with varying intensities of infection. Four *Crocidura* samples contained more than one *Eimeria* species/morphotypes. All three examined *Su. murinus* individuals were infected with *Eimeria* (Table 2).

**Table 2:** Microscopically detected coccidia-positive individuals/total examined eulipotyphlans and their origin.

	*Crocidura* spp.	*Neomys* spp.	*Sorex* spp.	*Suncus murinus*
Austria	1/1	2/3	0/2	0/0
Bulgaria	3/4	0/0	0/0	0/0
Czech Republic	2/8	5/17	7/90	0/0
Finland	0/0	0/0	1/9	0/0
France	0/1	0/0	0/1	0/0
Hungary	5/10	0/0	0/0	0/0
Italy	5/5	0/0	0/0	0/0
Kazakhstan	0/0	0/0	0/6	0/0
Latvia	0/0	2/2	1/1	0/0
Lithuania	0/0	0/1	1/7	0/0
Macedonia	1/1	0/0	0/0	0/0
Romania	1/1	0/0	0/0	0/0
Russia	0/0	1/1	0/0	0/0
Serbia	1/2	0/0	1/2	0/0
Slovakia	2/4	0/0	0/6	0/0
Taiwan	0/0	0/0	0/0	3/3
**Total**	**21/37**	**10/24**	**11/124**	**3/3**

Successful PCR amplification was achieved from 12 samples containing *Eimeria* spp. from Crocidurinae (5 *C. leucodon*, 2 *C. suaveolens*, 1 *C. russula*, 2 *Crocidura* spp., and 2 *Su. murinus*), 7 samples containing *Eimeria* spp. from Soricinae (2 *Sorex araneus*, 1 *N. anomalus*, 3 *N. fodiens*, and 1 *Neomys* sp.), and 2 samples containing *Isospora* spp. from *So. araneus* (Tables S1 and  S2). All these samples contained single-species infections, with the exception of sample B4B2, in which two distinct oocyst morphotypes were observed. Morphological and morphometrical analyses of oocysts were performed on 16 samples (unsporulated and damaged oocysts were excluded from measurement) (Table 1), and photomicrographs were taken from 12 samples (Fig. 2). In total, 50 sequences were obtained from the coccidia-positive samples (18 sequences of COX1, 15 sequences of COX3, and 17 sequences of 18S rRNA). Of these, all three genes were successfully sequenced from 10 samples (B4B2, 6HB1, 8HB1, 19CR, 34RUM, 54STR, RL, NF58, SA136, and TW4), and sequences of two genes were obtained from an additional nine samples (DK2, 6HB3, 23696, 23706, 60R, NF18, TW1, 16SM, and 21032). Only a single gene (COX1 and 18S rRNA) was successfully sequenced from two samples (35/13 and SA144, respectively).

**Figure 3:**
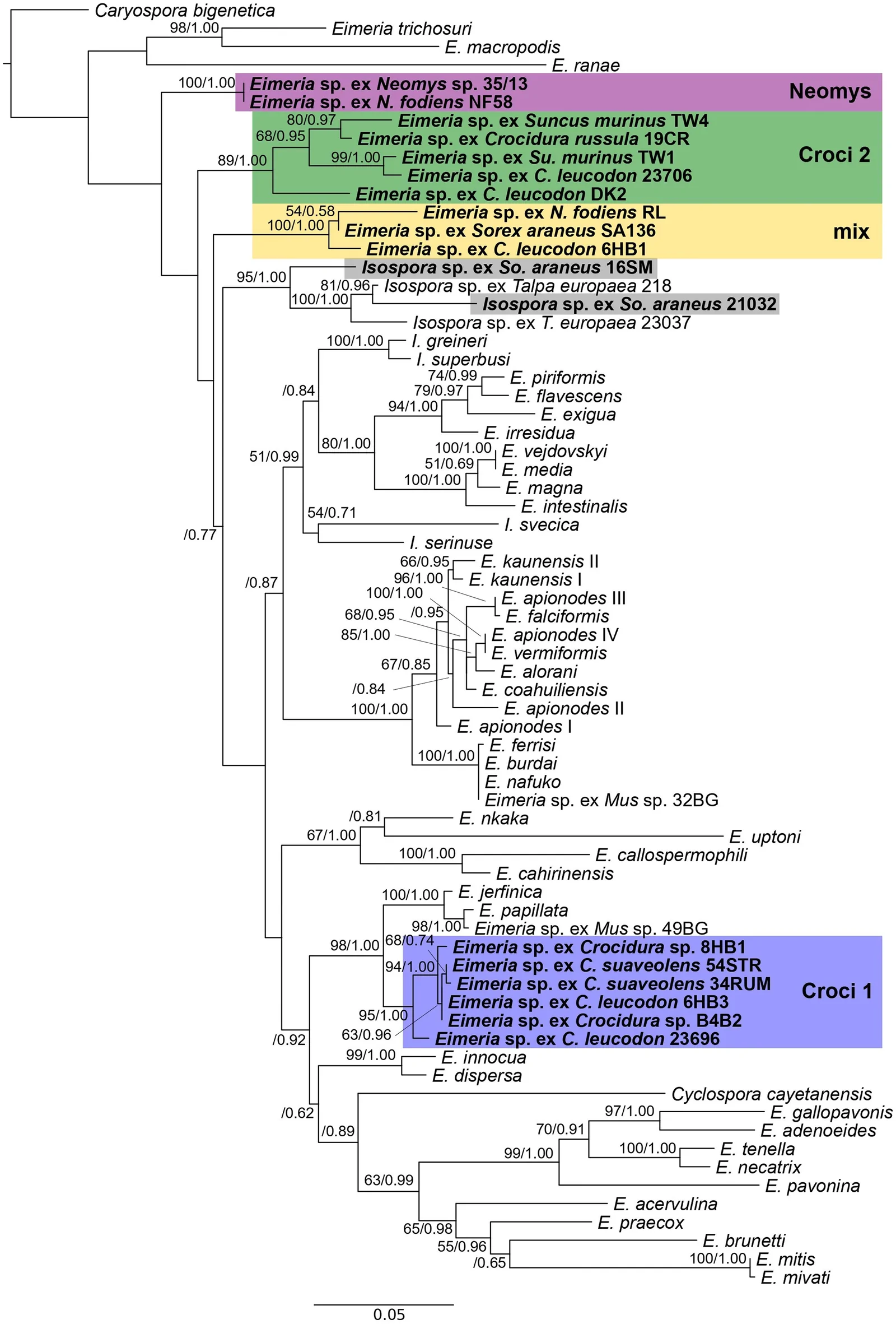
Phylogenetic relationships inferred from the ML analysis of the COX1 sequences of coccidia. The BI tree was mapped to the ML tree. Numbers at the nodes show bootstrap values derived from ML analysis/posterior probabilities under the BI analysis. Only bootstrap supports and posterior probabilities higher than 50% or 0.50, respectively, are displayed. Bootstrap supports and posterior probabilities lower than 50% or 0.50, respectively, are marked with dash (-). Taxa and their isolates in the tree are named as they are in the GenBank database. The tree is roooted with *Caryospora bigenetica*. The scale bar represents substitutions per site.

**Figure 4:**
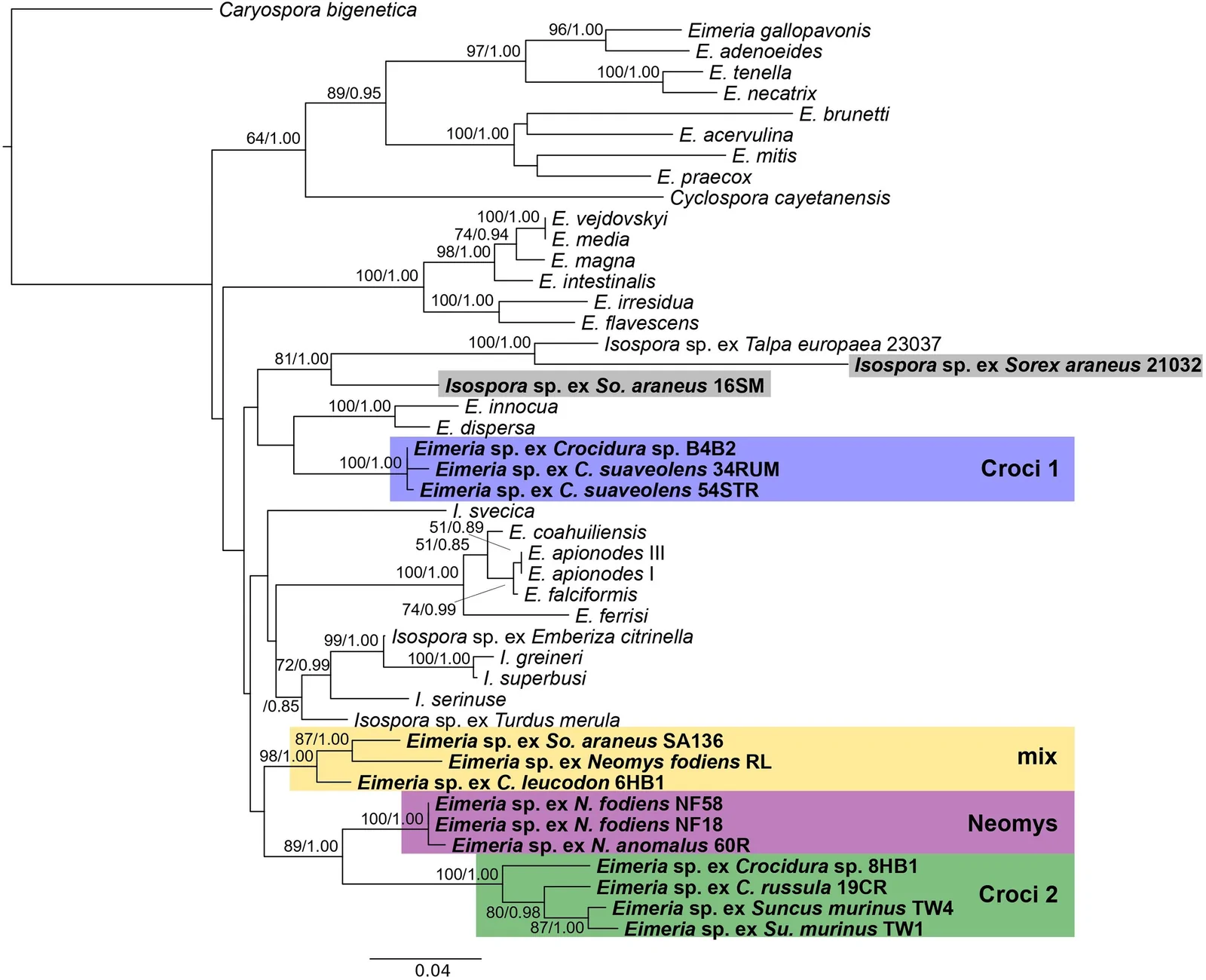
Phylogenetic relationships inferred from the ML analysis of the COX3 sequences of coccidia. The BI tree was mapped to the ML tree. Numbers at the nodes show bootstrap values derived from ML analysis/posterior probabilities under the BI analysis. Only bootstrap supports and posterior probabilities higher than 50% or 0.50, respectively, are displayed. Bootstrap supports and posterior probabilities lower than 50% or 0.50, respectively, are marked with dash (-). Taxa and their isolates in the tree are named as they are in the GenBank database. The tree is roooted with *Caryospora bigenetica*. The scale bar represents substitutions per site.

**Figure 5:**
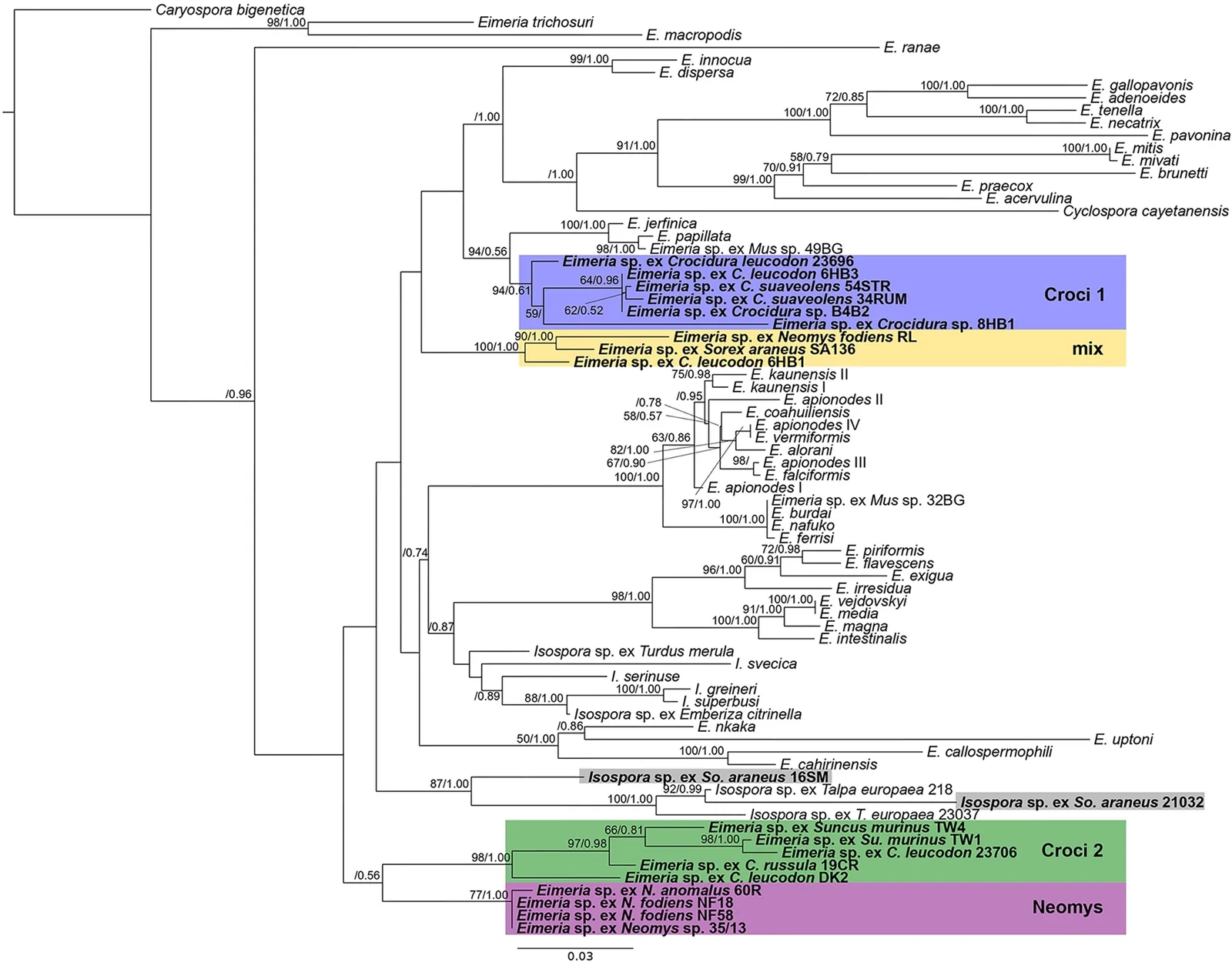
Phylogenetic relationships inferred from the ML analysis of the concatenate of COX1 and COX3 sequences of coccidia. The BI tree was mapped to the ML tree. Numbers at the nodes show bootstrap values derived from ML analysis/posterior probabilities under the BI analysis. Only bootstrap supports and posterior probabilities higher than 50% or 0.50, respectively, are displayed. Bootstrap supports and posterior probabilities lower than 50% or 0.50, respectively, are marked with dash (-). Taxa and their isolates in the tree are named as they are in the GenBank database. The tree is roooted with *Caryospora bigenetica*. The scale bar represents substitutions per site.

**Figure 6:**
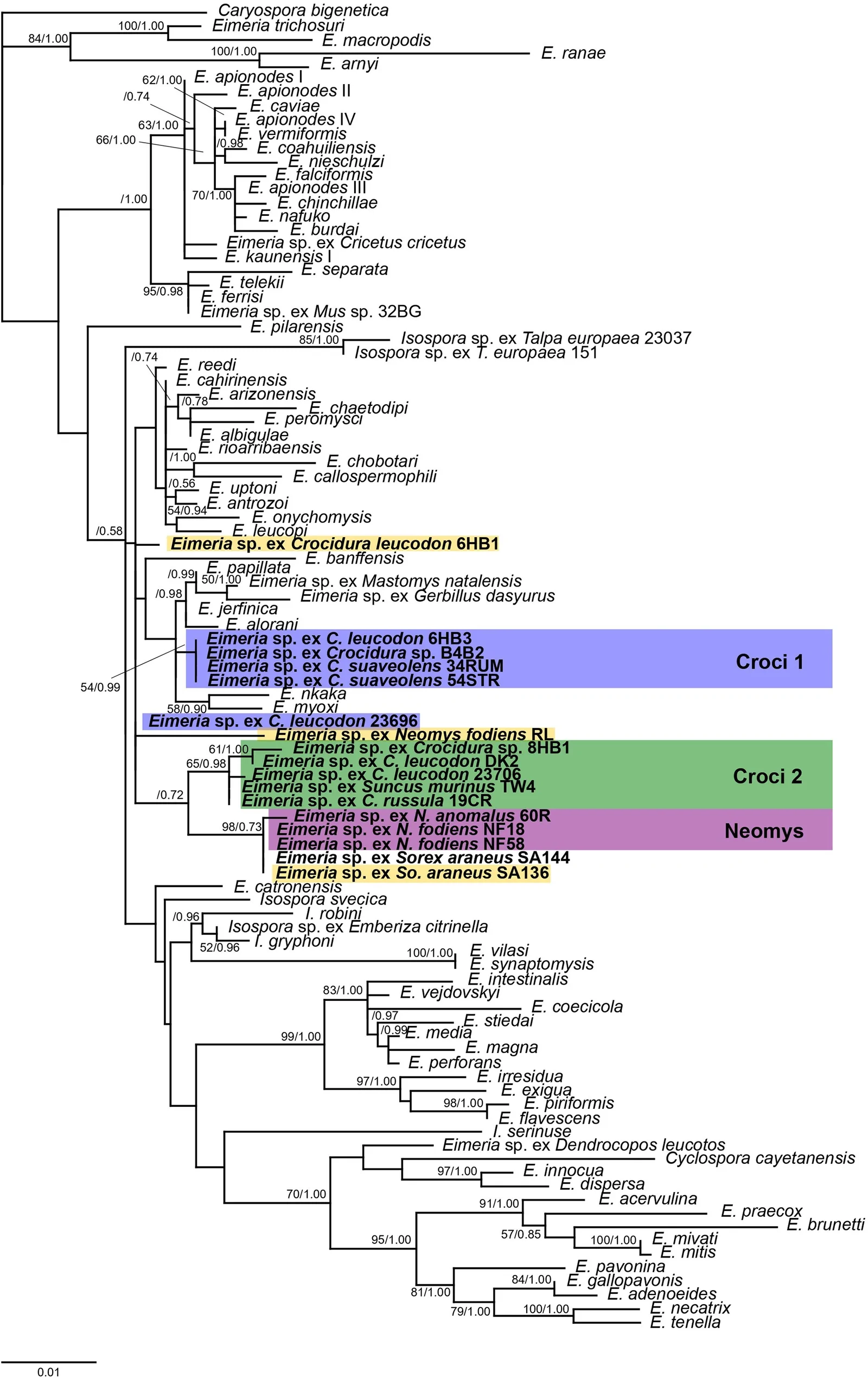
Phylogenetic relationships inferred from the ML analysis of the 18S rRNA sequences of coccidia. The BI tree was mapped to the ML tree. Numbers at the nodes show bootstrap values derived from ML analysis/posterior probabilities under the BI analysis. Only bootstrap supports and posterior probabilities higher than 50% or 0.50, respectively, are displayed. Bootstrap supports and posterior probabilities lower than 50% or 0.50, respectively, are marked with dash (-). Taxa and their isolates in the tree are named as they are in the GenBank database. The tree is roooted with *Caryospora bigenetica*. The scale bar represents substitutions per site.

Phylogenetic analyses of the COX1 and COX3 genes, as well as their concatenate, yielded well-resolved trees with a strongly supported inner structure (Figs 3–5). The 18S rRNA trees (Fig. 6) were compatible in their main branching with the COX1 and COX3 trees; however, they were less resolved. Sequences of eimerians from insectivores formed distinct clusters (Figs 3–6). One cluster was specific only to the genus *Crocidura* (designated here as “Croci1”), and it was conserved in analyses of all three genes. A second cluster (“Croci2”) was subfamily-specific, including samples from Crocidurinae—i.e. the genera *Crocidura* and *Suncus*. Samples originating from *Neomys* hosts formed the “*Neomys*” cluster, which was a sister cluster to “Croci2” in all phylogenetic analyses. In the 18S rRNA analyses, these *Neomys* samples grouped together with sample SA136 from *So. araneus*, which in both cytochrome analyses was placed in another cluster designated “Mix”, named after its composition of samples from different host genera. This cluster included samples SA136 from *So. araneus*, RL from *Neomys* sp., and 6HB1 from *C. leucodon* (Figs 3-6). Sequences of *Isospora* sp. from *So. araneus* clustered with isosporans from moles (Figs 3–5).

Only 16 samples were available for morphological and morphometrical analysis, and oocysts from eight of them were either partially sporulated or unsporulated (see Table 1). Morphological characteristics were compared with the phylogenetic placement of the corresponding sequences. No consistent morphological features correlating with phylogenetic clusters were observed. The sequences forming the “Croci1” cluster originated from samples corresponding to a single morphotype.

## Discussion

Mainly single-species infections, together with relatively low overall prevalence and infection intensities of coccidia were recorded in shrews, which is consistent with previous reports by Milek and Seville (2003) and Lynch and Duszynski (2008). In contrast, studies on another insectivorous mammal—the mole—have shown the opposite pattern, with mixed infections and a high prevalence of coccidia (almost 100%) (Duszynski and Wattam 1988). The exact cause of such a great difference in prevalence of coccidia between shrews and moles remains unclear. Hertel and Duszynski (1987) and Lynch and Duszynski (2008) hypothesized that it may be attributed to ecological, genetic, and physiological factors of the hosts (e.g. differences between shrews and moles in their metabolism, range size, time spent on the ground surface, etc.). It is possible that moles, which spend most of their lives underground (i.e. in a confined environment), can easily become infected with coccidia, and may maintain and spread the parasites in their burrows.

In free-ranging animals, low intensities of coccidian infections have been observed relatively frequently, and may result from several ecological and host-related factors. Acquired host immunity can limit parasite replication and reduce oocyst shedding, although immunity is generally not sterile and low numbers of oocysts may still be excreted by previously exposed animals. In addition, free-ranging hosts may experience lower and less continuous exposure to infective oocysts than animals maintained at high population densities. In captive populations, high host density, stress, repeated exposure and environmental accumulation of oocysts can facilitate parasite transmission, leading to frequent infections but low parasite burdens in individual hosts (Daugschies and Najdrowski 2005, Hecker et al. 2025).

Nevertheless, we consider it unlikely that the low numbers of coccidian oocysts observed were primarily attributable to the freshness or preservation quality of the samples. *Eimeria* oocysts possess a highly resistant wall that protects their internal structures from mechanical and chemical damage and enables them to persist in the environment for prolonged periods (Belli et al. 2006, Mai et al. 2009). Although degradation of host tissues may affect the quality of some samples, the robust oocyst wall makes complete degradation of oocyst structures relatively unlikely. Moreover, immediately after collection, all samples were preserved in 2.5% potassium dichromate solution, which is commonly used for the preservation and sporulation of coccidian oocysts. Williams et al. (2010) demonstrated that oocyst walls and internal structures of *Eimeria* remained well-preserved after almost 25 years of storage in 2.5% potassium dichromate solution. Thus, potassium dichromate preservation generally allows long-term maintenance of oocyst morphology. Nevertheless, some oocysts may have been too old, damaged, or otherwise unsuitable for successful sporulation; therefore, detailed morphological analyses could not be performed for all samples.

The morphological identification of *Eimeria* in shrews remains challenging. The original morphological descriptions of oocysts are often insufficient (being incomplete and/or of a poor quality), and the measurements provided in the published descriptions are sometimes not informative enough for reliable identification. For example, the oocyst size of *Eimeria dissimillis*, originally described from *So. araneus*, ranges from 18 to 33 × 13 to 24 µm (Yakimoff and Gousseff 1935). This range encompasses the size of nearly all the oocysts detected in the present study. In addition, the morphological plasticity of eimerian oocysts also makes identification based solely on size problematic. It is also possible that in this study, so far undescribed *Eimeria* species were observed. The lack of molecular data further complicates identification, as only a few sequences of eimerians from shrews are currently available in the GenBank database, and these are not assigned to species.

Another complication arises from synonymy. Confusion between *Eimeria* species and their synonyms is relatively common. Hertel and Duszynski (1987), who reviewed the literature on shrew eimerians, suggested that both *E. ropotomae* and *E. neomyi* (together with *E. komareki*) are junior synonyms of *E. chagasi*. However, in its original description, *E. chagasi* was reported to have a smooth oocyst wall, to lack a Stieda body, and to possess smaller oocysts than those of *E. ropotomae*. Based on the phylogenies obtained in this study, although it is not possible to determine the particular species with certainty, it is evident that highly morphologically similar oocysts cluster at different positions within the phylogenetic trees (Figs 3–6). Therefore, the three *Eimeria* species listed above may represent valid and distinct species, unless it is a polyphyletic taxon. Similar doubts regarding the validity of these species were also raised later by Duszynski and Upton (2000), who emphasized that, without clear knowledge of host specificity, these species should not be synonymized.

Confusion concerning synonymous names has often arisen from the assumption that *Eimeria* species are strictly host-specific and that host specificity can therefore serve as a reliable criterion for distinguishing species (Joyner 1982). However, very strict specificity limited only to a single host species appears to be relatively uncommon in *Eimeria*; more frequently, species are able to infect several hosts within a single genus. Some species have even been reported to cross generic or higher taxonomic boundaries—e.g. *E. apionodes*, which can infect *Apodemus, Microtus*, and *Myodes* rodents (Mácová et al. 2018), or *E. chinchillae*, which is capable of infecting members of multiple rodent families (De Vos 1970). It has also been documented that *Eimeria* species from one host may switch to a syntopically occurring host species (Trefancová et al. 2021). Information on the degree of host specificity of coccidia infecting insectivores is, however, limited. Nevertheless, several cases have been reported in which coccidia crossed even generic boundaries. For example, *Isospora brevicauda*, whose type host is *Blarina brevicauda* (Soricomorpha: Soricidae), was found in *Sorex monticolus*, and later also in *So. caecutiens, So. cinereus*, and *So. hoyi* (Milek and Seville 2003, Lynch and Duszynski 2008). The findings observed in this study suggest that some species are likely to be highly host-specific (the clades “Croci1”, “Croci2”, and “*Neomys*”), whereas others appear to have a broader host range (the clade “Mix”).

In this study, the limited number of samples available for morphological analysis, together with the frequent overlap of morphological characteristics among samples (Table 1), prevented the definition of distinct morphotypes, as has been done in previous publications (e.g. Mácová et al. 2018, Trefancová et al. 2021). Unlike rodent *Eimeria* species, coccidia from insectivores—aside from a few exceptions, including eimerians infecting hedgehogs and short-tailed shrews (genus *Blarina*)—generally lack an OR. This was also the case for the isolates examined in this study. Therefore, phylogenetic clustering based on the presence or absence of an OR is not relevant for coccidia from insectivores. Similarly, no consistent clustering patterns were observed in relation to other oocyst structures, such as the sporocyst residuum or polar granule/s.

The oscillation hypothesis proposes that parasites oscillate between phases of expansion, during which they exploit new host species, followed by a subsequent retraction, when only a few host species are infected. Consequently, speciation is thought to be driven from a more generalist ancestor, with occasional expansions to novel hosts (Janz et al. 2006, 2016). The phylogenies obtained in this study revealed the presence of several distinct phylogenetic lineages of *Eimeria* in shrews. We suggest that these lineages may have undergone such oscillations, exhibiting phylogenetic diversity but morphological similarity. However, due to the limited availability of comprehensive morphological and corresponding molecular data, precise evolutionary conclusions cannot yet be drawn.

Phylogenetic analyses revealed the presence of four distinct clusters (designated as “Croci1”, “Croci2”, “*Neomys*”, and “Mix”) (Figs 3–6). “Croci1” consisted of eimerians originating exclusively from *Crocidura* hosts. Based on morphology and morphometry, this cluster represented a single *Eimeria* species (Fig. 2), corresponding to *Eimeria neomyi*, originally described by Golemansky (1978) from *N. anomalus* and *N. fodiens* in Bulgaria (Table S3). The samples included in this cluster originated from *Crocidura* specimens collected in Bulgaria, the Czech Republic, and Romania, thereby sharing a geographic range with the type locality of *E. neomyi*.

The “Croci2” cluster was, similarly to “Croci1”, formed by eimerians from *Crocidura* hosts, with the addition of two samples from *Su. murinus*, a mammal belonging to the same subfamily. The morphological as well as sequential/phylogenetic similarities of these coccidians suggest that they may represent *Eimeria chagasi* or *E. neomyi*—species characterized by similar oocyst inner structures but differing in oocyst size—or *E. leucodontis*, which possesses a thicker oocyst wall. The oocysts in sample 23 706 resembled *E. chagasi*, but had a different sporocyst shape index, whereas those in samples 19CR, TW4, and 8HB1 corresponded to *E. neomyi* but possessed smaller sporocysts, or to *E. leucodontis*, which has a thicker oocyst wall (for more details, see Table S3). However, size is not a strict diagnostic feature, as both oocyst and sporocyst sizes may vary during the sporulation process and patency (Cheissin 1947, Duszynski 1971, Parker and Duszynski 1986). Size may also vary within a single *Eimeria* species infecting different host species (Reduker et al. 1985). Moreover, the reported size range of *E. chagasi* is quite broad (13–19 × 12–15 µm) (Yakimoff and Gousseff 1935; Table S3), encompassing the oocyst size observed across nearly the entire “Croci2” cluster. In addition, oocysts in samples DK2 and TW1 were unsporulated, which precluded morphological characterization and prevented their reliable assignment to a species or morphospecies.

The “*Neomys*” cluster comprised eimerian sequences obtained from *Neomys* hosts. Oocysts in sample NF58 morphologically corresponded to *E. chagasi*. Oocysts in sample 60R from *N. anomalus* were smaller than those in the other samples and were all unsporulated, similar to the oocysts from sample NF18; therefore, they could not be reliably identified based on morphology.

The “Mix” cluster was more diverse, containing samples obtained from three host genera—*Neomys* (RL), *Sorex* (SA136), and *Crocidura* (6HB1)—representing two distinct subfamilies, Soricinae and Crocidurinae. This indicates that at least some shrew-associated *Eimeria* species are not host-specific. Unfortunately, most of these samples contained unsporulated or damaged oocysts. The observed damage may have resulted from the thinness of the oocyst wall, which appeared fragile in all affected samples.

A single sample, 8HB1, clustered within the “Croci1” group in the COX1 and concatenated trees, but within the “Croci2” group in the COX3 and SSU trees. Such incongruent phylogenetic placement is not uncommon and can be interpreted in several ways. A likely scenario, frequently reported in *Eimeria* species, is hidden taxonomic diversity (intraspecific variability or the presence of cryptic species) (Mácová et al. 2018, Trefancová et al. 2021). Moreover, the sexual phase of the *Eimeria* life cycle provides an opportunity for genetic recombination, which may contribute to genetic diversity within populations. Alternatively, the incongruence may reflect natural variation among gene trees, as each locus has its own evolutionary history and may evolve at different rates.

Two obtained sequences of *Isospora* sp. from *Sorex* spp. clustered in both cytochrome analyses as well as in their concatenate with isosporan sequences from the European mole, *Talpa europaea*. The oocysts in sample 21032 morphologically corresponded to *Isospora araneae*, a species originally described from *So. araneus* by Golemansky (1978). Oocysts in sample 16SM were unsporulated; therefore, they could not be reliably identified based on morphology.

In general, the pathogenic effects of *Eimeria* have been documented primarily in domestic and farm animals, such as poultry, rabbits, or cattle (Daugschies and Najdrowski 2005, Pakandl 2009, Blake et al. 2021). In contrast, little is known about *Eimeria* infections in wild-living animals, including rodents, which represent the most extensively studied wild hosts of these coccidia. Infections in wild mammals are often considered to be of low pathogenicity, and overt clinical signs are rarely reported. However, virtually nothing is known about the pathogenicity or potential negative impact of the recorded eimerians on shrews. Such information would require histopathological examination of gastrointestinal tissues from infected individuals. As this study was based on dead-found specimens, it was not possible to include this approach. Nevertheless, a number of factors may influence the potential impact of these parasites on their hosts, including parasite load, degree of host specificity of the parasite species (whether they can infect also other species living in sympatry/syntopy), environmental persistence of oocysts, and the health status of the host individual.

Overall, the results indicate that the diversity of *Eimeria* infecting shrews is higher than previously recognized, and that both evolutionary and methodological factors contribute to the current taxonomic uncertainty. The occurrence of multiple phylogenetically distinct but morphologically similar lineages underscores that morphological traits alone are insufficient for reliable species identification. It is also necessary to consider frequent host switches—particularly among hosts living in syntopy—as well as the presence of cryptic species and intraspecific variability. Additionally, unresolved synonymies and the limited availability of molecular data further obscure species boundaries. Future studies integrating detailed morphological characterization with multilocus molecular analyses and broader host sampling will be essential to clarify the taxonomy, host specificity, and evolutionary history of *Eimeria* in insectivores.

## Supplementary Material

xtag047_Supplemental_Files

## Data Availability

The nucleotide sequences generated in this study have been deposited in the GenBank database under the accession numbers provided in the manuscript (Supplementary Table S1). All data generated or analyzed during this study are included in this published article and its Supplementary Materials.

## References

[bib1] Bangoura B, Bhuyia MAI, Kilpatrick M. *Eimeria* infections in domestic and wild ruminants with reference to control options in domestic ruminants. Parasitol Res. 2022;121:2207–32. 10.1007/s00436-022-07564-x. 35680677

[bib2] Barta JR, Martin DS, Liberator PA. Phylogenetic relationships among eight *Eimeria* species infecting domestic fowl inferred using complete small subunit ribosomal DNA sequences. J Parasitol. 1997;83:262–71. 10.2307/3284453. 9105308

[bib3] Belli SI, Smith NC, Ferguson DJP. The coccidian oocyst: a tough nut to crack! Trends Parasitol. 2006;22:416–23. 10.1016/j.pt.2006.07.004. 16859995

[bib4] Berto BP, McIntosh D, Lopes CWG. Studies on coccidian oocysts (Apicomplexa: eucoccidiorida). Rev Bras Parasitol Vet. 2014;23:1–15. 10.1590/S1984-29612014001. 24728354

[bib5] Blake DP, Marugan-Hernandez V, Tomley FM. Spotlight on avian pathology: *eimeria* and the disease coccidiosis. Avian Pathol. 2021;50;209–13. 10.1080/03079457.2021.1912288. 33823695

[bib6] Cheissin EM. Mutability of the oocysts of *Eimeria magna*. Zool Zhurn. 1947;26:17–29.

[bib7] Daugschies A, Najdrowski M. Eimeriosis in cattle: current understanding. J Vet Med Ser B. 2005;52:417–27. 10.1111/j.1439-0450.2005.00894.x. 16364016

[bib8] De Vos AJ. Studies on the host range of *Eimeria chinchillae* De Vos & Van der Westhuizen, 1968. Onderstepoort J Vet Res. 1970;37:29–36. 5526336

[bib9] Duszynski DW. Increase in size of *Eimeria separata* oocysts during patency. J Parasitol. 1971;57:948–52. 10.2307/3277841. 5133901

[bib10] Duszynski DW, Eckerlin RP, McCarthy TJ. *Eimeria* species from *Cryptotis* shrews (Insectivora: Soricidae) with description of a new species. J Parasitol. 2003;89:974–7. 10.1645/GE-3135. 14627146

[bib11] Duszynski DW, Marquardt WC. Coccidia in the mammary glands of shrews (Order: insectivora). J Parasitol. 2003;89:609–11. 10.1645/GE-3141RN. 12880266

[bib12] Duszynski DW, Upton SJ. Coccidia (Apicomplexa: Eimeriidae) of the Mammalian Order Insectivora. Special publication of the Museum of South western Biology No. 4. Albuquerque: University of New Mexico Press, 2000.

[bib13] Duszynski DW, Upton SJ. Cyclospora, Eimeria, Isospora, and Cryptosporidium spp. In: Samuel WM, Pybus MJ, Kocan AA (eds.), Parasitic Diseases of wild Mammals, 2nd edn. Iowa: Iowa State Press, 2001, 416–33. 10.1002/9780470377000 10.1002/9780470377000.ch16c.

[bib14] Duszynski DW, Wattam AR. Coccidian parasites (Apicomplexa: eimeriidae) from insectivores. IV. Four new species in *Talpa europaea* from England. J Protozool. 1988;35:58–62. 10.1111/j.1550-7408.1988.tb04077.x. 3367321

[bib15] Duszynski DW, Wilber PG. A guideline for the preparation of species descriptions in the Eimeriidae. J Parasitol. 1997;83:333–36. 10.2307/3284470. 9105325

[bib16] Gardner SL, Duszynski DW. Polymorphism of eimerian oocysts can be a problem in naturally infected hosts: an example from subterranean rodents in Bolivia. J Parasitol. 1990;76:805–11. 10.2307/3282798. 2254815

[bib17] Golemansky V. Description of 9 new species of coccidia (Coccidia Eimeriidae), parasites of small mammals, in Bulgaria. Acta Protozool. 1978;17:261–70.

[bib18] Golemansky V, Yankova P. Studies on the species composition and occurrence of coccidia (Sporozoa, Coccidia) in some small mammals in Bulgaria. Bull Inst Zool Mus. 1973;37:5–31.

[bib19] Gouy M, Guindon S, Gascuel O. SeaView version 4: a multiplatform graphical user interface for sequence alignment and phylogenetic tree building. Mol Biol Evol. 2010;27:221–4. 10.1093/molbev/msp259. 19854763

[bib20] Guindon S, Gascuel O. A simple, fast, and accurate algorithm to estimate large phylogenies by maximum likelihood. Syst Biol. 2003;52:696–704. 10.1080/10635150390235520. 14530136

[bib21] Hecker AS, Raulf M-K, König S et al. Individual host factors and co-infections affect the probability and excretion intensity of endoparasite infections in dairy cows. Parasites Vectors. 2025;18:311. 10.1186/s13071-025-06974-x. 40739254 PMC12309137

[bib22] Hertel LA, Duszynski DW. Coccidian parasites (Apicomplexa: eimeriidae) from insectivores. III. Seven new species in shrews (Soricidae: soricinae) from Canada, Japan, and the United States. J Parasitol. 1987;73:172–83. 10.2307/3282363. 3572649

[bib23] Hinsu AT, Thakkar JR, Koringa PG et al. Illumina Next Generation Sequencing for the analysis of *Eimeria* populations in commercial broilers and indigenous chickens. Front Vet Sci. 2018;5:176. 10.3389/fvets.2018.00176. 30105228 PMC6077195

[bib24] Huelsenbeck JP, Ronquist F. MRBAYES: bayesian inference of phylogenetic trees. Bioinformatics. 2001;17:754–5. 10.1093/bioinformatics/17.8.754. 11524383

[bib25] Janz N, Braga MP, Wahlberg N et al. On oscillations and flutterings—a reply to Hamm and Fordyce. Evolution. 2016;70:1150–5. 10.1111/evo.12927. 27094253

[bib26] Janz N, Nylin S, Wahlberg N. Diversity begets diversity: host expansions and the diversification of plant-feeding insects. BMC Evol Biol. 2006;6:4. 10.1186/1471-2148-6-4. 16420707 PMC1382262

[bib27] Joyner LP. Host and Site Specificity. In: Long PL (ed.), The Biology of the Coccidia. Baltimore, MD: University Park Press, 1982, 35–62.

[bib28] Katoh K, Standley DM. MAFFT multiple sequence alignment software version 7: improvements in performance and usability. Mol Biol Evol. 2013;30:772–80. 10.1093/molbev/mst010. 23329690 PMC3603318

[bib29] Kearse M, Moir R, Wilson A et al. Geneious Basic: an integrated and extendable desktop software platform for the organization and analysis of sequence data. Bioinformatics. 2012;28:1647–9. 10.1093/bioinformatics/bts199. 22543367 PMC3371832

[bib30] Kreier JP, Baker JR. Parasitic Protozoa. London: Allen and Unwin, 1987. 10.1007/978-94-011-6847-2.

[bib31] Kubiski SV, Witte C, Burchell J et al. Mitochondrial gene diversity and host specificity of *Isospora* sequences in passerine birds. Front Vet Sci. 2022;9:847030. 10.3389/fvets.2022.847030. 35847651 PMC9280662

[bib32] Kvičerová J, Hypša V. Host-parasite incongruences in rodent *Eimeria* suggest significant role of adaptation rather than cophylogeny in maitenance of host specificity. PLoS One. 2013;8:e63601. 23861732 10.1371/journal.pone.0063601 PMC3701668

[bib33] Kvičerová J, Mikeš V, Hypša V. Third lineage of rodent eimerians: morphology, phylogeny and re-description of *Eimeria myoxi* (Apicomplexa: emeriidae) from *Eliomys quercinus* (Rodentia: gliridae). Parasitology. 2011;138:1217–23. 21810299 10.1017/S0031182011001107

[bib34] Kvičerová J, Pakandl M, Hypša V. Phylogenetic relationships among *Eimeria* spp. (Apicomplexa, Eimeriidae) infecting rabbits: evolutionary significance of biological and morphological features. Parasitology. 2008;135:443–52. 18248685 10.1017/S0031182007004106

[bib35] Long PL, Joyner LP. Problems in the identification of species of *Eimeria*. J Protozool. 1984;31:535–41. 10.1111/j.1550-7408.1984.tb05498.x. 6392531

[bib36] Lynch AJ, Duszynski DW. Species of coccidia (Apicomplexa: eimeriidae) in shrews from Alaska, U.S.A., and Northeastern Siberia, Russia, with description of two new species. J Parasitol. 2008;94:883–8. 10.1645/GE-1506.1. 18576829

[bib37] Mácová A, Hoblíková A, Hypša V et al. Mysteries of host switching: diversification and host specificity in rodent-coccidia associations. Mol Phylogenet Evol. 2018;127:179–89. 10.1016/j.ympev.2018.05.009. 29753710

[bib38] Mai K, Sharman PA, Walker RA et al. Oocyst wall formation and composition in coccidian parasites. Mem Inst Oswaldo Cruz. 2009;104:281–9. 10.1590/S0074-02762009000200022. 19430654

[bib39] Matsubayashi M, Takami K, Niichiro A et al. Molecular characterization of crane Coccidia, *Eimeria gruis* and *E. reichenowi*, found in feces of migratory cranes. Parasitol Res. 2005;97:80–83. 10.1007/s00436-005-1404-9. 15940517

[bib40] McAllister CT, Hnida JA, Kinsella JM. *Eimeria longirostris* (Apicomplexa: eimeriidae) from Vagrant Shrew, *Sorex vagrans* (Mammalia: eulipotyphla: Soricidae), from Montana, USA. Acta Parasit. 2021;66:710–3. 10.1007/s11686-020-00321-z. 33392957

[bib41] Milek JA, Seville S. Species of *Eimeria* and *Isospora* (Apicomplexa: eimeriidae) from shrews (Insectivora: Soricidae) in Northwestern Wyoming, U.S.A. Comp Parasitol. 2003;70:72–77. 10.1654/1525-2647(2003)070[0072:SOEAIA]2.0.CO;2.

[bib42] Morrison DA, Bornstein S, Thebo P et al. The current status of the small subunit rRNA phylogeny of the coccidia (Sporozoa). Int J Parasitol. 2004;34:501–14. 10.1016/j.ijpara.2003.11.006. 15013740

[bib43] Ogedengbe ME, Qvarnstrom Y, da Silva AJ et al. A linear mitochondrial genome of *Cyclospora cayetanensis* (Eimeriidae, Eucoccidiorida, Coccidiasina, Apicomplexa) suggests the ancestral start position within mitochondrial genomes of eimeriid coccidia. Int J Parasitol. 2015;45:361–5. 10.1016/j.ijpara.2015.02.006. 25812835 PMC4610128

[bib44] Page RDM. TREEVIEW: an application to display phylogenetic trees on personal computers. Comput Applic Biosci. 1996;12:357–58. 10.1093/bioinformatics/12.4.357 8902363

[bib45] Pakandl M. Coccidia of rabbit: a review. Folia Parasitol. 2009;56:153–66. 10.14411/fp.2009.019. 19827358

[bib46] Parker BB, Duszynski DW. Polymorphism of eimerian oocysts: a dilemma posed by working with some naturally infected hosts. J Parasitol. 1986;72:602–4. 10.2307/3281518. 3783354

[bib47] Posada D. jModelTest: phylogenetic model averaging. Mol Biol Evol. 2008;25:1253–6. 10.1093/molbev/msn083. 18397919

[bib48] Posada D. Selection of models of DNA evolution with jModelTest. Methods Mol Biol. 2009;537:93–112. 10.1007/978-1-59745-251-9 10.1007/978-1-59745-251-9_5. 19378141

[bib49] Power ML, Richter C, Emery S et al. *Eimeria trichosuri*: phylogenetic position of a marsupial coccidium, based on 18S rDNA sequences. Exp Parasitol. 2009;122:165–8. 10.1016/j.exppara.2009.02.008. 19248779

[bib50] Reduker DW, Hertel L, Duszynski DW. *Eimeria* species (Apicomplexa: Eimeriidae) infecting *Peromyscus* rodents in the Southwestern United States and Northern Mexico with description of a new species. J Parasitol. 1985;71:604–13. 10.2307/3281432. 4057004

[bib51] Schwarz RS, Jenkins MC, Klopp S et al. Genomic analysis of *Eimeria* spp. populations in relation to performance levels of broiler chicken farms in Arkansas and North Carolina. J Parasitol. 2009;95:871–80. 10.1645/GE-1898.1. 20049993

[bib52] Smith PH, Seklau BS, Wiles E et al. Collection, preservation, and diagnostic methods. In: Baker DG (ed.), Flynn’s Parasites of Laboratory Animals, 2nd edn. Iowa: Wiley-Blackwell, 2007, 1–13. 10.1002/9780470344552 10.1002/9780470344552.ch1.

[bib53] Trefancová A, Kvičerová J, Mácová A et al. Switch, disperse, repeat: host specificity is highly flexible in rodent-associated *Eimeria*. Int J Parasitol. 2021;51:977–84. 10.1016/j.ijpara.2021.04.005. 34089715

[bib54] Trefancová A, Mácová A, Kvičerová J. Isosporan oocysts in the faeces of bank voles (*Myodes glareolus*; Arvicolinae, Rodentia): real parasites, or pseudoparasites?. Protist. 2019;170:104–20. 10.1016/j.protis.2018.12.002. 30738338

[bib55] Vrba V, Pakandl M. Coccidia of turkey: from isolation, characterisation and comparison to molecular phylogeny and molecular diagnostics. Int J Parasitol. 2014;44:985–1000. 10.1016/j.ijpara.2014.06.004. 25020103

[bib56] Vrba V, Pakandl M. Host specificity of turkey and chicken *Eimeria*: controlled cross-transmission studies and a phylogenetic view. Vet Parasitol. 2015;208:118–24. 10.1016/j.vetpar.2015.01.017. 25660426

[bib57] Williams RB, Thebo P, Marshall RN et al. Coccidian oöcysts as type-specimens: long-term storage in aqueous potassium dichromate solution preserves DNA. Syst Parasitol. 2010;76:69–76. 10.1007/s11230-010-9234-2. 20401580

[bib58] Yakimoff WL, Gousseff WF. On the coccidia of shrews, grass-snakes, and lizards. J R Microsc Soc. 1935;55:170–3. 10.1111/j.1365-2818.1935.tb04403.x.

[bib59] Zajac AM, Conboy GA. Veterinary Clinical Parasitology. Iowa: Blackwell Publishing, 2006.

[bib60] Zhao X, Duszynski DW. Phylogenetic relationships among rodent *Eimeria* species determined by plastid ORF470 and nuclear 18S rDNA sequences. Int J Parasitol. 2001;31:715–9. 10.1016/S0020-7519(01)00136-9. 11336753

