# Supplementary material for: Phylogeny of coccidian parasites (Eimeriorina, Apicomplexa) infecting shrews
Source: FEMS Microbes. 2026 Sep 4;7:xtag047. doi: 10.1093/femsmc/xtag047 (PMC13573310; doi:10.1093/femsmc/xtag047)
Supplement: Supplementary file 1 [file xtag047_supplemental_files.zip › Table S1_F.docx]

Supplementary Table S1. Taxa and sequences included in the phylogenetic analyses.

| Taxon | Accession number COX1 | Accession number COX3 | Accession number SSU |
| --- | --- | --- | --- |
| *Caryospora bigenetica* | KP658102 | KP658102 | AF060975 |
| *Cyclospora cayetanensis* | KP231180 | KP231180 | AF111183 |
| *Eimeria acervulina* | FJ236419 | KNA094948 | U67115 |
| *Eimeria adenoeides* | KJ608415 | KJ608415 | AF324212 |
| *Eimeria albigulae* | na | na | AF307880 |
| *Eimeria alorani* | KU215486 | na | JQ993659 |
| *Eimeria antrozoi* | na | na | AF307876 |
| *Eimeria apionodes* I | MT934376 | MH698513 | MT921433 |
| *Eimeria apionodes* II | KU215482 | na | JQ993658 |
| *Eimeria apionodes* III | KU215468 | MH698516 | KU174470 |
| *Eimeria apionodes* IV | KU215940 | na | KU192935 |
| *Eimeria arizonensis* | na | na | AF307878 |
| *Eimeria arnyi* | na | na | AY613853 |
| *Eimeria banffensis* | na | na | JQ993644 |
| *Eimeria brunetti* | KX094959 | KX094959 | KT184337 |
| *Eimeria burdai* | JQ993709 | na | JQ993666 |
| *Eimeria cahirinensis* | JQ993686 | na | JQ993645 |
| *Eimeria callospermophili* | JQ993688 | na | JQ993648 |
| *Eimeria catronensis* | na | na | AF324213 |
| *Eimeria caviae* | na | na | JQ993649 |
| *Eimeria chaetodipi* | na | na | AF339489 |
| *Eimeria chinchillae* | na | na | JQ993650 |
| *Eimeria chobotari* | na | na | AF324214 |
| *Eimeria coahuiliensis* | MT934398 | MH698530 | MT921437 |
| *Eimeria coecicola* | na | na | EF694015 |
| *Eimeria dispersa* | KJ608416 | KJ608416 | HG793041 |
| *Eimeria exigua* | JQ993691 | na | HQ173829 |
| *Eimeria falciformis* | KX495129 | KX495129 | AF080614 |
| *Eimeria ferrisi* | MH777558 | na | MH752036 |
| *Eimeria flavescens* | KP025693 | KP025693 | EF694011 |
| *Eimeria gallopavonis* | KJ608417 | KJ608417 | KT184343 |
| *Eimeria innocua* | KR108296 | KR108296 | HG793045 |
| *Eimeria intestinalis* | KP009592 | KP009592 | KF856284 |
| *Eimeria irresidua* | KP025690 | KP025690 | HQ173832 |
| *Eimeria jerfinica* | KU215518 | na | KU192971 |
| *Eimeria kaunensis* I | KU215536 | na | JQ993662 |
| *Eimeria kaunensis* II | KU215453 | na | na |
| *Eimeria leucopi* | na | na | AF339491 |
| *Eimeria macropodis* | JQ392580 | na | JQ392575 |
| *Eimeria magna* | KF419217 | KF419217 | EF694016 |
| *Eimeria media* | KP025691 | KP025691 | HQ173834 |
| *Eimeria mitis* | KC409031 | KC409031 | FR775307 |
| *Eimeria* cf. *mivati* | EF174185 | na | FJ236378 |
| *Eimeria myoxi* | na | na | JF304148 |
| *Eimeria nafuko* | JQ993708 | na | JQ993665 |
| *Eimeria necatrix* | KX094954 | KX094954 | U67119 |
| *Eimeria nieschulzi* | na | na | U40263 |
| *Eimeria nkaka* | JQ993697 | na | JQ993651 |
| *Eimeria onychomysis* | na | na | AF307879 |
| *Eimeria papillata* | KT184377 | na | KT184350 |
| *Eimeria pavonina* | JN596590 | na | JN596589 |
| *Eimeria perforans* | na | na | EF694017 |
| *Eimeria peromysci* | na | na | AF339492 |
| *Eimeria pilarensis* | na | na | AF324215 |
| *Eimeria piriformis* | JQ993698 | na | EF694014 |
| *Eimeria praecox* | KX094945 | KX094945 | FJ236362 |
| *Eimeria ranae* | KU524488 | na | EU717219 |
| *Eimeria reedi* | na | na | AF311642 |
| *Eimeria rioarribaensis* | na | na | AF307877 |
| *Eimeria separata* | na | na | AF311643 |
| *Eimeria* sp. ex *Cricetus cricetus* | na | na | JQ993663 |
| *Eimeria* sp. ex *Dendrocopos leucotos* DAM-2009 | na | na | FN298443 |
| *Eimeria* sp. ex *Gerbillus dasyurus* | na | na | JQ993664 |
| *Eimeria* sp. ex *Mastomys natalensis* | na | na | JQ993667 |
| *Eimeria* sp. ex *Mus* sp. 32BG | KU255439 | na | KU314657 |
| *Eimeria* sp. ex *Mus* sp*.* 49BG | KU255440 | na | na |
| *Eimeria stiedai* | na | na | HQ173837 |
| *Eimeria synaptomysis* | na | na | JQ993652 |
| *Eimeria telekii* | na | na | AF246717 |
| *Eimeria tenella* | KX094951 | KX094951 | JNA093898 |
| *Eimeria trichosuri* | JN192136 | na | FJ829322 |
| *Eimeria uptoni* | KU216004 | na | KU192941 |
| *Eimeria vejdovskyi* | KP025692 | KP025692 | HQ173838 |
| *Eimeria vermiformis* | MH777577 | na | KT184355 |
| *Eimeria vilasi* | na | na | JQ993653 |
| *Isospora greineri* | KR108298 | KR108298 | na |
| *Isospora gryphoni* | na | na | AF080613 |
| *Isospora robini* | na | na | AF080612 |
| *Isospora serinuse* | KX276860 | KX276860 | na |
| *Isospora* sp. ex *Apodemus flavicollis* B13 | na | na | na |
| *Isospora* sp. ex *Emberiza citrinella* | na | MK573849 | MK559089 |
| *Isospora* sp. ex *Serinus canaria* RY-2015a | na | na | KR477877 |
| *Isospora* sp. ex *Talpa europaea* 23037 | MN184728 | MN184734 | MN149909 |
| *Isospora* sp. ex *Talpa europaea* 151 | na | na | JQ993670 |
| *Isospora* sp. ex *Talpa europaea* 218 | JQ993714 | na | na |
| *Isospora* sp. ex *Turdus merula* | na | MK573850 | na |
| *Isospora superbusi* | KT203396 | KT203396 | na |
| *Isospora svecica* | MK573841 | MK573853 | MK559092 |
|  |  |  |  |
| *Eimeria* sp. ex *Crocidura* sp. B4B2 | **MF589745** | **MG839069** | **MG770469** |
| *Eimeria* sp. ex *Crocidura* sp. 8HB1 | **MF589744** | **MG839071** | **MG770468** |
| *Eimeria* sp. ex *Crocidura leucodon* DK2 | **MF589751** | **na** | **MG770462** |
| *Eimeria* sp. ex *C. leucodon* 6HB1 | **MF589752** | **MG839060** | **MG770466** |
| *Eimeria* sp. ex *C. leucodon* 6HB3 | **MG779411** | **na** | **MG770467** |
| *Eimeria* sp. ex *C. leucodon* 23696 | **MF589748** | **na** | **MG770463** |
| *Eimeria* sp. ex *C. leucodon* 23706 | **MF589749** | **na** | **MG770464** |
| *Eimeria* sp. ex *Crocidura russula* 19CR | **MG779412** | **MG839072** | **MG770471** |
| *Eimeria* sp. ex *Crocidura suaveolens* 34RUM | **MF589750** | **MG839063** | **MG770470** |
| *Eimeria* sp. ex *C. suaveolens* 54STR | **MF589747** | **MG839064** | **MG770465** |
| *Eimeria* sp. ex *Neomys* sp*.* 35/13 | **MG779414** | **na** | **na** |
| *Eimeria* sp. ex *Neomys* sp*.* RL | **MG779413** | **MG839070** | **MG770478** |
| *Eimeria* sp. ex *Neomys anomalus* 60R | **na** | **MG839066** | **MG770472** |
| *Eimeria* sp. ex *Neomys fodiens* NF18 | **na** | **MG839061** | **MG770473** |
| *Eimeria* sp. ex *N. fodiens* NF58 | KU255438 | **MG839065** | **MG770474** |
| *Eimeria* sp. ex *Sorex araneus* SA136 | JQ993710 | **MG839067** | **MG770476** |
| *Eimeria* sp. ex *So. araneus* SA144 | **na** | **na** | **MG770477** |
| *Eimeria* sp. ex *Suncus murinus* TW1 | **MN184723** | **MN184729** | **na** |
| *Eimeria* sp. ex *Su. murinus* TW4 | **MN184724** | **MN184730** | **MN149906** |
| *Isospora* sp. ex *So. araneus* 16SM | **MN184725** | **MN184731** | **na** |
| *Isospora* sp. ex *So. araneus* 21032 | **MF589746** | **MG839062** | **na** |

Sequences obtained in this study are highlighted in bold. *E*. *apionodes* I-IV and *E*. *kaunensis* I-II are labelled according to Mácová et al. (2018) and Trefancová et al (2021).

na = the sequence is not available.
