# Supplementary material for: Phylogeny of coccidian parasites (Eimeriorina, Apicomplexa) infecting shrews
Source: FEMS Microbes. 2026 Sep 4;7:xtag047. doi: 10.1093/femsmc/xtag047 (PMC13573310; doi:10.1093/femsmc/xtag047)
Supplement: Supplementary file 1 [file xtag047_supplemental_files.zip › Table S2_F.docx]

Supplementary Table S2. Information on sequenced samples from shrews.

| **Sample code** | **Host species** | **Locality and date of collection** |
| --- | --- | --- |
| B4B2 | *Crocidura* sp. | BG, Kamen Brjag, 30.8.2014 |
| 8HB1 | *Crocidura* sp. | HU, 20.9.2013 |
| DK2 | *Crocidura leucodon* | CZ, Třísov/Dívčí Kámen, 11.9.2015 |
| 6HB1 | *C. leucodon* | HU, Medgyesegyháza, 18.9.2013 |
| 6HB3 | *C. leucodon* | HU, Medgyesegyháza, 18.9.2013 |
| 23696 | *C. leucodon* | SK, Hrhov, 29.9.2016 |
| 23706 | *C. leucodon* | SK, Hrhov, 29.9.2016 |
| 19CR | *Crocidura russula* | ES, La Roca del Valles, 26.9.2009 |
| 54STR | *Crocidura suaveolens* | CZ, Křečov, 20.11.2016 |
| 34RUM | *C. suaveolens* | RO, Carașova, 26.8.2016 |
| 35/13 | *Neomys* sp. | RU, Pskov, 28.9.2013 |
| 60R | *Neomys anomalus* | AT, Aigen, 18.9.2015 |
| NF18 | *Neomys fodiens* | CZ, Boršov nad Vltavou, 10.8.2007 |
| NF58 | *N. fodiens* | CZ, České Budějovice, 26.8.2007 |
| RL | *N. fodiens* | CZ, Litvínov, 13.10.2012 |
| SA136 | *Sorex araneus* | CZ, Boršov nad Vltavou - Březí, 15.9.2008 |
| SA144 | *So. araneus* | CZ, Záhorčice, 26.9.2008 |
| 16SM | *So. araneus* | CZ, Mořičov, 23.11.2018 |
| 21032 | *So. araneus* | SK, Bušince, 11.6.2008 |
| TW1 | *Suncus murinus* | TW, Gancheng, 8.11.2018 |
| TW4 | *Su. murinus* | TW, Gancheng, 9.11.2018 |

AT = Austria; BG = Bulgaria; CZ = Czech Republic; ES = Spain; HU = Hungary; RO = Romania; RU = Russia; SK = Slovakia; TW = Taiwan.
