# Supplementary material for: Phylogeny of coccidian parasites (Eimeriorina, Apicomplexa) infecting shrews
Source: FEMS Microbes. 2026 Sep 4;7:xtag047. doi: 10.1093/femsmc/xtag047 (PMC13573310; doi:10.1093/femsmc/xtag047)
Supplement: Supplementary file 1 [file xtag047_supplemental_files.zip › Table S3_F.docx]

Supplementary Table S3. Overview of *Eimeria* and *Isospora* spp. so far reported from shrew taxa.

| Species of coccidia | Author of description | Host | Origin | Oocyst | | | | | Sporocyst | | | | |
| --- | --- | --- | --- | --- | --- | --- | --- | --- | --- | --- | --- | --- | --- |
|  |  |  |  | **Shape** | **Size [um]** | **SI** | **Wall [um]** | **PG** | **Shape** | **Size [um]** | **SI** | **SB** | **SR** |
| *Eimeria* *chagasi* | Yakimoff and Gousseff, 1935 | SA, SM | BG, CZ, RU | SPH | 14.5 × 14.5 (13-19 × 12-15) | 1.0-1.1 | single-layered; 1 | + | na | 8-9 × 4-5 | na | 0 | + |
| *Eimeria darjeelingensis* * | Sinha and Sinha, 1980 | SuM | IN | SUB | 16.7 × 14.9 (15-19 × 13-18) | 1.1 | bilayered; 0.3 | - | OVO | 15 × 10 | 1.4 | + | + |
| *Eimeria* *dissimilis* | Yakimoff and Gousseff, 1935 | SA, SM | BG, LT, RU | ELO, OVO, pointed ends | 28 × 19.4 (18-33 × 13-24) | 1.4 | single-layered; 1 | + | ELI | 15 × 10 | na | 0 | 0? |
| *Eimeria* *leucodontis* | Musaev and Veisov, 1961 | CL, CS | BG, AZ | SPH | 21 × 21 (18-24 × 18-24) | 1.0 | bilayered; 2.5 | - | na | 8.6 × 6.4  (8-10 × 6-8) | 1.3 | 0 | + |
| *Eimeria murinus* * | Bandyopadhyay and Dasgupta, 1985 | SuM | IN | SUB | 18.8 × 16.1 (15-21 × 14-16.5) | 1.2 | bilayered; 1.2-1.5 | - | OVO | 12.6 × 7.1  (8.5-13.5 × 6.5-8) | 1.8 | + | + |
| *Eimeria* *neomyi* | Golemansky, 1978 | NA, NF | BG | SPH - SUB | 18-20 × 18-20 (for SPH);  18.3 × 16.5 (16-20.5 × 15.2-17.8) (for SUB) | 1.0; 1.1 | bilayered; 1 | + | na | 12 × 7.5 | 1.6 | + (hyaline plug) | + |
| *Eimeria* *ropotomae* | Golemansky, 1978 | CL | BG |  | 22-25 × 20-22.5 | 1.1 | bilayered?; sculptured;  >1 | +  (1-2) | OVO | 12 × 7.6 | 1.6 | + (hyaline plug) | + |
| *Eimeria* *soricis* | Henry, 1932 | SA, SM | LT, RU, USA | SUB, OVO, ELI | 19 × 14 (19-22 × 13-14) | 1.4 | single-layered; <1 | + | ELO - ELI | ? | ? | 0 | +? |
| *Eimeria suncus* | Ahluwalia et al., 1979 | SuM | IN | SUB | 19.5 × 15.2 (18-22 × 15.5-17) | 1.3 | bilayered; 1.7-1.9 | - | LEM | 10.5 × 7.8  (9.5-11 × 6.5-9) | 1.35 | + | + |
| *Eimeria tenrececaudata* | Couch et al., 2011 | AF, SuM | MG | SUB | 18.8 × 17.4 (17-22 × 15-20) | 1.1 | single-layered; ~1 | 0-2 | LEM | 9.9 × 6.6  (9-11 × 5-8) | 1.5 | + | + |
| *Isospora araneae* | Golemansky, 1978 | SA | BG | SPH - SUB | 17 × 17 (for SPH);  16-18.5 × 15-18 (for SUB) | 1.0 | bilayered; ~1 | +  (1-2) | OVO | 12.5 × 9.5 | 1.3 | 0? | + |
| *Isospora neomyi* | Golemansky, 1978 | NA, NF | BG | SPH | 12-16 × 12-16 | 1.0 | bilayered; ~1 | 1 | OVO | 8-11 × 5-6.5 | na | 0 | + |
| *Isospora soricis* * | Golemansky and Yankova, 1973 | SA | BG | ELI | 24 × 17 (23-25 × 16-18) | 1.5 | single-layered; <1 | 1 | SPH | 10.5 × 10.5 | 1.0 | 0 | + |

Abbreviations:

AF = Afrosoricomorpha; AZ = Azerbaijan; BG = Bulgaria; CL = *Crocidura leucodon*; CS = *Crocidura* *suaveolens*; CZ = Czech Republic; ELI = ellipsoidal; ELO = elongated; IN = India; LEM = lemon-shaped; LT = Lithuania; MG = Madagascar; NA = *Neomys anomalus*; na = data not available; NF = *Neomys* *fodiens*; OVO = ovoidal; PG = polar granule; RU = Russia; SA = *Sorex araneus*; SB = Stieda body; SI = shape index (length/width); SM = *Sorex* *minutus*; SPH = spheroidal; SR = sporocyst residuum; SUB = subspheroidal; SuM = *Suncus murinus*; USA = United States of America; + = present; - = absent.

* this species was described from a single animal individual and has not been recorded since its original description ([eimeria.unl.edu/list.html](http://eimeria.unl.edu/isoricis.html)).

References:

Ahluwalia SS, Singh RV, Arora GS, Mandal AK, Sarkar NC. *Eimeria suncus* sp. nov. (Sporozoa: Eimeriidae) from the common house shrew, *Suncus murinus murinus* Linnaeus. *Acta Protozool* 1979;*18*:451-54.

Bandyopadhyay S, Dasgupta B. A new coccidium, *Eimeria murinus*, from *Suncus murinus murinus* (Linnaeus) in West Bengal, India. *Indian J Parasitol* 1985;*9*:101-03**.**

Couch L, Laakkonen J, Goodman S, Duszynski DW. Two new eimerians (Apicomplexa) from insectivorous mammals in Madagascar. *J Parasitol* 2011;*97*: 293-96.

Golemansky V. Description of 9 new species of coccidia (Coccidia Eimeriidae), parasites of small mammals, in Bulgaria. *Acta Protozool* 1978;*17*:261-70.

Golemansky V, Yankova P. Studies on the species composition and occurrence of coccidia (Sporozoa, Coccidia) in some small mammals in Bulgaria. *Bull Inst Zool Mus* 1973;*37*:5-31.

Henry DP. Observations on the coccidia of small mammals in California, with descriptions of seven new species. *Univ Calif Publ Zool* 1932;*37*:279-90.

Musaev MA, Veisov AM. New coccidium species from *Crocidura leucodon* Herm. *Dokl Akad Nauk Azerbaid* 1961;*17*:967-69.

Sinha CK, Sinha S. *Eimeria darjeelingensis* sp. n. from a house-shrew, *Suncus murinus soccatus* (Hodgson). *Acta Protozool* 1980;*19*:293-96.

Yakimoff WL, Gousseff WF. On the coccidia of shrews, grass-snakes, and lizards. *J R Microsc Soc* 1935;*55*:170-73.
